# Performance of a novel protease-activated fluorescent imaging system for intraoperative detection of residual breast cancer during breast conserving surgery

**DOI:** 10.1007/s10549-021-06106-w

**Published:** 2021-02-21

**Authors:** Conor R. Lanahan, Bridget N. Kelly, Michele A. Gadd, Michelle C. Specht, Carson L. Brown, Kevin S. Hughes, Rong Tang, Upahvan Rai, Elena F. Brachtel, Travis Rice-Stitt, Barbara L. Smith

**Affiliations:** 1 Division of Surgical Oncology, Massachusetts General Hospital, MGH Center for Breast Cancer, Yawkey 9A, 55 Fruit St., Boston, MA 02114 USA; 2 Department of Pathology, Massachusetts General Hospital, Boston, MA USA

**Keywords:** Image-guided surgery, Lumpectomy, Intraoperative tumor detection, Margin assessment

## Abstract

**Purpose:**

Safe breast cancer lumpectomies require microscopically clear margins. Real-time margin assessment options are limited, and 20–40% of lumpectomies have positive margins requiring re-excision. The LUM Imaging System previously showed excellent sensitivity and specificity for tumor detection during lumpectomy surgery. We explored its impact on surgical workflow and performance across patient and tumor types.

**Methods:**

We performed IRB-approved, prospective, non-randomized studies in breast cancer lumpectomy procedures. The LUM Imaging System uses LUM015, a protease-activated fluorescent imaging agent that identifies residual tumor in the surgical cavity walls. Fluorescent cavity images were collected in real-time and analyzed using system software.

**Results:**

Cavity and specimen images were obtained in 55 patients injected with LUM015 at 0.5 or 1.0 mg/kg and in 5 patients who did not receive LUM015. All tumor types were distinguished from normal tissue, with mean tumor:normal (*T*:*N*) signal ratios of 3.81–5.69. *T*:*N* ratios were 4.45 in non-dense and 4.00 in dense breasts (*p* = 0.59) and 3.52 in premenopausal and 4.59 in postmenopausal women (*p* = 0.19). Histopathology and tumor receptor testing were not affected by LUM015. Falsely positive readings were more likely when tumor was present < 2 mm from the adjacent specimen margin. LUM015 signal was stable in vivo at least 6.5 h post injection, and ex vivo at least 4 h post excision.

**Conclusions:**

Intraoperative use of the LUM Imaging System detected all breast cancer subtypes with robust performance independent of menopausal status and breast density. There was no significant impact on histopathology or receptor evaluation.

## Introduction

Breast cancer remains among the most common malignancies in women. Lumpectomy and mastectomy provide equivalent survival [1, 2], but a safe lumpectomy requires microscopically tumor-free margins to reduce risk of local recurrence and death [3–5]. Unfortunately, real-time margin assessment options are limited, and as high as 20–40% of lumpectomy patients require a second surgical procedure to correct positive margins [6–13].

New tools for intraoperative margin assessment are needed. In addition to meeting requirements for safety, sensitivity and specificity, an intraoperative margin assessment tool for breast cancer surgery must work within existing clinical and laboratory system constraints. It should perform well across the full range of breast cancer histological types and patient characteristics. It should not interfere with standard preoperative or intraoperative workflow. There should be no impact on standard histopathology or receptor testing.

We have studied the LUM Imaging System for real-time, intraoperative margin assessment during breast cancer lumpectomy surgery. Fluorescent signal is generated and detected at sites of tumor in the surgical bed and in excised specimens using (1) LUM015, a novel PEGylated protease-activated far-red fluorescent imaging agent [14]; (2) the LUM optical head, a hand-held probe used to excite LUM015 and collect real-time fluorescent recordings of the lumpectomy cavity [15]; and (3) software for image analysis. In an initial pilot study, we established protocols for use of the system and showed that its use was feasible within standard perioperative workflow [16]. Areas of fluorescence could be identified in lumpectomy cavity walls and excised.

We previously reported results of a feasibility trial of the LUM Imaging System in breast conserving surgery [17]. Use of the system in 45 breast cancer lumpectomy patients with invasive breast cancer or ductal carcinoma in situ (DCIS) showed 84% sensitivity and 73% specificity for intraoperative tumor detection. The system is also being evaluated for use in surgery for peritoneal (NCT03834272), central nervous system (NCT03717142), and prostate (NCT03441464) cancers.

We now evaluate the performance of the LUM Imaging System across patient and breast tumor types and assess its impact on histopathology evaluation and preoperative and intraoperative workflow.

## Materials and methods

We conducted an IRB-approved, prospective, non-randomized, open-label study of the Lumicell (LUM) Imaging System (Lumicell, Newton, MA) in breast cancer patients undergoing lumpectomy at the Massachusetts General Hospital, Boston, MA (NCT02438358). The LUM Imaging System includes LUM015, a novel PEGylated protease-activated far-red fluorescent imaging agent [14]; the LUM optical head, a hand-held probe, used to excite LUM015 and collect real-time fluorescent recordings [15]; and software for image analysis to identify regions of suspected residual cancer. The LUM Imaging System excites activated LUM015 with a 630-nm LED source. A charge coupled device (PCO AG, Germany) collects fluorescent photons after they pass through an emission filter and an imaging lens. Fluorescent signal values are reported in a 10^10^ counts/s/cm^2^. We used the 2.6 cm diameter LUM optical head, which has a 5.3 cm^2^ area, circular field of view and is covered by a sterile plastic sleeve for intraoperative use.

Eligibility requirements and study cohorts have been previously described [16]. Briefly, patients undergoing lumpectomy for ductal carcinoma in situ (DCIS) or invasive breast cancer received IV LUM015 at 0.5 or 1.0 mg/kg 2–6 h prior to surgery in the perioperative unit. Study investigators and institutional research nurses were designated to perform the 3-min injection via peripheral IV. All surgical procedures were performed by 3 dedicated surgeons (BLS, MAG, and MCS). After a standard lumpectomy procedure (including specimen imaging for marker localized non-palpable tumors and removal of additional tissue at any positive margins identified on specimen imaging, by palpation or inspection), cavity walls were imaged with the LUM Imaging System probe in vivo, and shaved margins 0.5–1.0 cm thick were removed from the cavity per standard institutional practice. Excised lumpectomy and shaved cavity margin specimen were also imaged ex vivo prior to specimen margin inking. Tumor:normal (*T*:*N*) fluorescent signal ratios were determined by transecting ex vivo lumpectomy specimens and comparing signal produced by tumor versus adjacent normal tissue. A higher *T*:*N* ratio indicates greater contrast between tumor and normal tissue. Ratios were in the 2.5–5 range in prior studies. All specimen processing was performed per institutional standards with analysis by dedicated breast pathologists [18–20].

All specimens had unique tumor-to-surface distance measurements evaluated in each orientation for correlation with LUM Imaging System results. For invasive cancers, specimen margins were considered positive if invasive tumor or DCIS was present at the outer margin, or on ink. For DCIS without invasion, margins were positive if DCIS was present < 2 mm from ink.

Background breast tissue fluorescence was measured in excised lumpectomy specimens of 20 patients who did not receive LUM015 but had various combinations of sentinel node mapping agents. Measurements were obtained in 5 patients in each category: no sentinel node mapping, Technetium-99 (Tc-99) sulfur colloid alone, Tc-99 with isosulfan blue and Tc-99 with methylene blue. LUM Images were taken directly following specimen excision and fluorescent data was captured with LUM Image acquisition.

Ten patients had serial measurements of total complement, histamine, and tryptase levels prior to administration of 1.0 mg/kg of LUM015 and at 15, 30, and 60 min after injection for safety assessments.

Statistical analyses were performed using a Student’s two-tailed t-test assuming heteroscedasticity when comparing two independent variables and a one-way Analysis of Variance (ANOVA) when comparing more than two independent variables.

## Results

### Patient population

55 patients received intravenous LUM015, 5 at 0.5 mg/kg and 50 at 1.0 mg/kg doses. Median patient age was 60 (range 44–79) years and mean BMI was 27.5 (range 20.4–44.4) kg/m^2^. All patients had breast conserving surgery for biopsy-confirmed invasive or intraductal cancer. Tumors included 31 (56.4%) invasive ductal carcinomas (IDC) with or without ductal carcinoma in situ (DCIS), 5 (9.1%) invasive lobular carcinomas (ILC), 5 (9.1%) invasive cancers with ductal and lobular features, and 14 (25%) DCIS without invasion. Thirty-nine (71%) patients were postmenopausal and 16 were pre- or perimenopausal. Mammographic breast density varied from almost entirely fatty to extremely dense, with 62% of breasts classified as heterogeneously or extremely dense (Table 1).

**Table 1:** Patient demographics (*n* = 55)

Median age (years)	60 (44–79)
Mean BMI (kg/m^2^) (range)	27.5 (20.4–44.4)
LUM015 injection dose	
0.5 mg/kg	5 (9%)
1.0 mg/kg	50 (91%)
Cancer histological type^a^	
Invasive ductal carcinoma ± DCIS	31 (56.4%)
Invasive lobular carcinoma	5 (9.1%)
Invasive with ductal and lobular features	5 (9.1%)
Pure DCIS	14 (25.4%)
Breast density on preoperative mammogram	
Almost entirely fatty	2 (4%)
Scattered areas of fibroglandular density	19 (34%)
Heterogeneously dense	29 (53%)
Extremely dense	2 (4%)
Mixed scattered fibroglandular/heterogeneously dense	3 (5%)
Race	
White	46 (84%)
Black	5 (9%)
Asian	2 (4%)
Unknown	2 (4%)
Menopause status	
Postmenopausal	39 (71%)
Premenopausal/perimenopausal	16 (29%)

*BMI* body mass index, *DCIS* ductal carcinoma in situ

^a^One patient had both an invasive ductal and an invasive lobular cancer

### Implementing the system into standard surgical workflow

Use of the LUM Imaging System did not significantly interfere with preoperative or intraoperative workflow. LUM015 was administered intravenously over 3 min, 56–402 min prior to intraoperative imaging of the lumpectomy cavity. This did not significantly affect patient arrival time to the hospital, as most patients were routinely scheduled to arrive at least 2 h before the scheduled start of surgery, and many have arrived earlier to accommodate sentinel node isotope injection or marker localization of non-palpable tumors. LUM015 could be injected before or after isotope injection or wire/tag localization of non-palpable lesions.

Prior to use, the imaging probe was draped with a sterile barrier by a scrub tech, with an average of 2.33 (range 1–5) minutes required for draping. Barrier application routinely occurred during room setup, prior to the start of the procedure. Intraoperatively, the device displayed a live feed image of the in vivo tissue surface as the surgeon moved the imaging probe along the lumpectomy cavity walls. Initialization of the device required 6 representative images of the cavity. Average cavity initialization time was 48.5 (range 25–94) s. The tumor detection software required less than one second to display highlighted areas of suspected residual cancer on the live feed. The speed of the software and 2.6 cm diameter view of the optical head allowed the surgeon to rapidly image the entire cavity. Cavity walls could be re-imaged after resection of areas of fluorescent signal to verify removal of all high signal areas.

Cavity imaging did not significantly lengthen surgical procedures or delay specimen delivery to the pathology lab. Mean total time required for intraoperative cavity imaging was 6.78 (range 0.83–19.13) minutes in the single center feasibility study and 6.67 (range 0.62–23) minutes in the initial multicenter feasibility study. These imaging times include time spent performing initial cavity imaging, time spent excising additional margin specimens based on device readings, repeat imaging after removal of additional margin tissue, and included each surgeon’s initial training cases with the system. Among 16 patients with no suspicious LUM015 cavity signal who did not require additional margin resection, mean total imaging time was 2.67 (range 0.83–7.20) minutes.

### Impact on histopathology processing

Use of LUM Imaging System did not affect results of standard histopathology, immunohistochemistry (IHC) or fluorescent in situ hybridization (FISH) analyses. We compared estrogen receptor (ER), progesterone receptor (PR) and human epidermal growth factor receptor 2 (HER2) values from the initial diagnostic core biopsy with values obtained from the lumpectomy specimen excised after administration of LUM015 and intraoperative cavity imaging. No patient’s HER2 status changed after administration of LUM015. Rates of concordance between ER and PR readings from core biopsy to excised lumpectomy samples were similar to that of other series (Table 2) [21]. No residual LUM015 fluorescence was detected on fluorescence microscopy imaging of an unstained, formalin fixed, paraffin embedded lumpectomy tissue specimen from a study patient.

**Table 2:** Concordance of ER, PR and HER2 measurements on core biopsy versus lumpectomy specimens

Receptor	Lumicell trial		Asogan et al. [18]	
	# Concordant/total tested	Concordance rate (%)	# Concordant/total tested	Concordance rate (%)
ER	34/35	98	259/264	98
PR	29/33	88	235/262	89
HER2 (IHC and/or FISH)	37/37	100	453/468	97

*ER* estrogen receptor, *PR* progesterone receptor, *HER2* human epidermal growth factor receptor 2, *IHC* immunohistochemistry, *FISH* fluorescent in situ hybridization

### System performance by tumor histology

The effectiveness of this system depends on its ability to produce and detect fluorescent signal that distinguishes tumor from surrounding normal tissue. To address system performance across a variety of tumor types and patient characteristics, transected lumpectomy specimens from 23 patients were imaged to capture 34 images containing both tumor and normal tissue. We also determined whether the LUM015 tumor-to-normal (*T*:*N*) fluorescence ratio was lower in tumors with smaller cross-sectional volume, such as DCIS or areas of invasive lobular cancer.

The system was able to distinguish tumor from normal tissue for all breast cancer histological types, with mean *T*:*N* ratios of 3.81 for IDC ± DCIS, 3.98 for ILC ± DCIS and 5.69 for specimens with only DCIS, *p* = 0.25 (Table 3). The small cross-sectional area of DCIS-filled ducts and the single file growth pattern of ILC produced *T*:*N* signal ratios that were not significantly different from those of the more common IDC lesions. There was no difference in tumor signal (*p* = 0.22) or adjacent normal tissue signal (*p* = 0.42) among histological types.

**Table 3:** Tumor-to-normal fluorescence ratios for different tumor histological types

Tumor histology	*n*	Average normal fluorescence		Average tumor fluorescence		Average tumor:normal signal ratio	
DCIS	7	4.56	*p* = 0.42^a^	16.06	*p* = 0.22^a^	5.69	*p* = 0.25^a^
IDC	23	4.29		13.91		3.81	
ILC	4	2.48		10.31		3.98	

*DCIS* Ductal carcinoma in situ, *IDC* invasive ductal cancer, *ILC* invasive lobular cancer

^a^Calculated with a one-way analysis of variance ANOVA

### System performance by breast density and menopausal status

We evaluated the impact of mammographic breast density and patient menopausal status on fluorescence levels and system performance. We evaluated *T*:*N* LUM015 signal ratios in patients with lower breast density (fatty or scattered fibroglandular densities) and in patients with dense breasts (heterogeneously or extremely dense). The system distinguished tumor from normal tissue equally well for low density (*T*:*N*  = 4.45) and dense breasts (*T*:*N*  = 4.00, *p* = 0.59). However, both tumor signal and normal tissue background signal were significantly higher (*p* = 0.03 and *p* < 0.01, respectively) for dense breasts compared with non-dense breasts (Table 4).

**Table 4:** Tumor-to-normal fluorescence ratios by mammographic breast density and patient menopausal status

	*n*	Average normal fluorescence		Average tumor fluorescence		Average tumor:normal signal ratio	
Breast density							
Almost entirely fatty or scattered areas of fibroglandular density	16	3.08	*p* < 0.01^a^	12.10	*p* = 0.03^a^	4.45	*p* = 0.59^a^
Heterogeneously dense or extremely dense	18	5.06		15.55		4.00	
Menopausal status							
Pre- or perimenopausal	12	4.33	*p* = 0.74^a^	13.94	*p* = 0.99^a^	3.52	*p* = 0.19^a^
Postmenopausal	22	4.02		13.92		4.59	

^a^Calculated with a 95% confidence interval, two-tailed Student’s *t*-test assuming heteroscedasticity

Patient menopausal status did not affect system performance. Tumor signal (*p* = 0.99), adjacent background signal (*p* = 0.74) and *T*:*N* ratios (*p* = 0.19) did not differ significantly between pre- and perimenopausal versus postmenopausal women (Table 4).

### Variation in LUM015 signal based on tumor distance from imaged surface

**Fig. 1:**
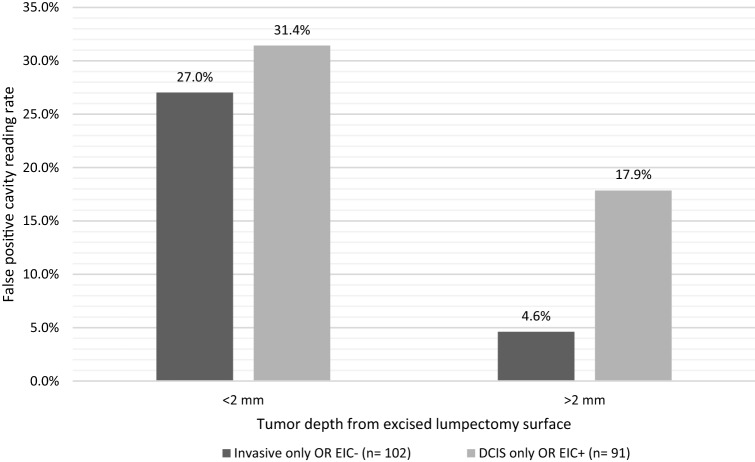
Effect of close lumpectomy margins on false-positive LUM015 cavity readings. *DCIS* ductal carcinoma in situ, *EIC* extensive intraductal component with DCIS within and beyond the invasive tumor

We previously noted that shaved margin specimens taken from some areas of LUM015 signal did not contain tumor on standard histopathology analysis [16, 17]. We hypothesized that these falsely positive readings were more frequent in areas immediately adjacent to tumor, reflecting a gradient of LUM015 activation by proteases present at the periphery of malignant lesions. To assess this, we looked at rates of falsely positive LUM015 signal at cavity surfaces adjacent to excised specimens with tumor ≤ 2 mm vs. > 2 mm from the excised specimen edge. When invasive cancers without an extensive intraductal component (EIC) were ≤ 2 mm from the edge of the excised specimen, the rate of falsely positive LUM015 signal at the adjacent cavity surface was 27%, compared with 4.6% when invasive cancer was > 2 mm from the excised specimen edge. For tumors with only DCIS or for EIC-positive invasive cancers, the rate of falsely positive LUM015 signal at the adjacent cavity surface was 31% when DCIS was ≤ 2 mm from the edge of the excised specimen but fell to 18% when DCIS was > 2 mm from the excised specimen edge (Fig. 1).

### Variation in LUM015 signal over time

We previously reported that adequate LUM015 activation for intraoperative detection required a minimum of 101 min between LUM015 injection and intraoperative imaging and remained robust for at least 402 min after injection [16, 17]. We evaluated the durability of ex vivo tumor:normal fluorescent signal ratios as a function of time after specimen excision. Specimens were imaged 76–246 min after excision and all maintained tumor:normal fluorescent signal ratios greater than 1.97, with no apparent decrement in signal over this time period. These data suggest that the LUM015 signal is durable ex vivo as well as in vivo.

### Safety assessment

**Fig. 2:**
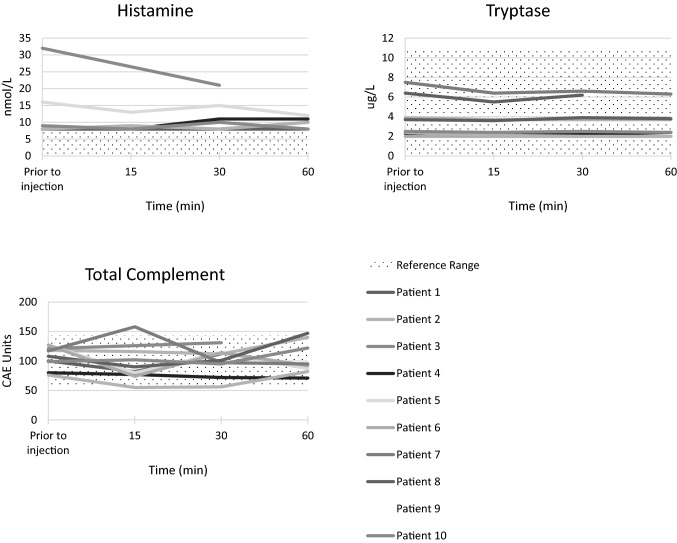
Histamine, tryptase and complement levels over time after LUM015 injection

After injection with LUM015, patients had blue-colored urine for 24–48 h as the dye was excreted, but no other common side effects were noted. There were no clinically significant differences between preoperative and 3–6-week postoperative complete blood count, serum chemistry or liver function test values in patients who received LUM015 injections. One patient who received LUM015 in a separate clinical trial had an anaphylactic reaction following injection but recovered completely [22]. We evaluated the impact of LUM015 on total complement, histamine, and tryptase levels prior to injection and at 15, 30 and 60 min after injection in 10 patients who received LUM015. We found no significant changes in the levels of any of these mediators after injection of LUM015 (Fig. 2).

### Evaluation of breast tissue autofluorescence and impact of sentinel node mapping agents

Isosulfan blue and methylene blue dyes, which are injected into the breast for sentinel node mapping, fluoresce under wavelengths of light similar to those used to excite LUM015. To assess compatibility of node mapping agents with LUM015, we measured background autofluorescence of breast tissue and fluorescence after injection of Technetium-99 sulfur colloid (Tc-99), isosulfan blue, and methylene blue in excised lumpectomy specimens of 20 patients who did not receive LUM015 injection.

Average normal breast tissue background autofluorescence (no LUM015, blue dye or Tc-99) was 2.18 ± 0.96 × 10^10^ counts/s/cm^2^. After Tc-99 injection, normal tissue fluorescent signal was 2.24 ± 1.44 × 10^10^ counts/s/cm^2^, not significantly different from the control (*p* = 0.42). Both isosulfan blue and methylene blue created very high background signal, 67.97 ± 26.99 and 24.78 ± 14.91 × 10^10^ counts/s/cm^2^, respectively, in areas of visible blue tissue staining. Signal was high even in areas with no visible blue discoloration on the tissue surface, 21.52 ± 26.83 × 10^10^ counts/s/cm^2^ for isosulfan blue and 8.55 ± 9.22 × 10^10^ counts/s/cm^2^ for methylene blue. For comparison, average in vivo LUM015 tumor fluorescent signal was 10.95 × 10^10^ and 10.11 × 10^10^ counts/s/cm^2^ for 0.5 and 1.0 mg/kg LUM015 injections, respectively [16].

## Discussion

Options for real-time margin assessment during cancer surgery remain limited. For women with breast cancer, this results in positive margins that require a second or even third surgical procedure in up to 40% of lumpectomies [6–13]. Even when margins are negative by current histopathology standards, tumor is left behind at rates high enough that radiation is routinely required to reduce local recurrence to acceptable levels [1, 23]. Better options for rapid and accurate margin assessment are needed.

We previously reported that the LUM Imaging System showed 84% sensitivity and 73% specificity for detection of tumor in 570 cavity margin surfaces in 40 breast cancer patients [17]. Image analysis and display requires only 1 s per 2.6 cm diameter circular margin surface [16].

We now report additional data about the performance of the LUM Imaging System for intraoperative breast lumpectomy margin assessment. We found that tumor signal was robust for both invasive ductal and invasive lobular cancers, and for invasive cancers with mixed histology. Importantly, signal was also robust for DCIS, the extent of which may be underestimated on imaging, and is not palpable or visible intraoperatively. Tumor was distinguished from normal tissue in both dense and fatty breasts and for both pre- and postmenopausal women.

We also report that use of the system was feasible within routine preoperative and intraoperative workflow and added minimal time to the surgical procedure. The requirement for a minimum of 101 min between LUM015 injection and intraoperative imaging fit within the standard time that patients were asked to arrive at the hospital before surgery. LUM015 could be administered before or after isotope injection for sentinel node mapping and before or after marker localization of non-palpable tumors, increasing flexibility of integration into preoperative workflow. Set up for the imaging system and draping of the optical head averaged 2 min.

Intraoperatively, the system’s large field of view and rapid image acquisition allowed the entire lumpectomy cavity to be scanned in approximately 1 min, adding little time to the surgical procedure. The cavity could be re-scanned after removal of areas suspicious for residual tumor, to verify removal of all suspicious findings. Rapid assessment of the entire cavity is a key advantage of this system over standard histopathology, as standard histopathology evaluates less than 1% of the surface of an excised specimen.

This system also has the advantage of identifying the location of tumor in the cavity wall, rather than on the surface of a pliable excised specimen. We previously showed that standard pathology analysis of a specimen margin has only 51% specificity for predicting findings in a shaved margin from the corresponding cavity location [24].

In vivo LUM015 fluorescent signal was stable for at least 6.5 h after injection, and ex vivo signal was maintained for at least 4 h after excision. Durable in vivo signal shows that the system is not impacted by unplanned surgical delays and durable ex vivo signal confirms the reliability of ex vivo *T*:*N* signal ratio measurements. LUM015 did not impact standard histopathology processing or staining, and did not affect measurement of ER, PR and HER2 by immunohistochemistry or fluorescent in situ hybridization.

In some cavity margins, the system detected fluorescent signal suspicious for residual tumor, but no tumor was identified on standard histopathology testing of tissue from that site. We found that these falsely positive readings were more likely at sites where tumor was present < 2 mm from the adjacent specimen margin. We hypothesize that this reflects a gradient of LUM015 activation by proteases present at the periphery of malignant lesions. Although this property reduces the apparent specificity of the system, it may have the positive effect of helping to obtain clear margins of desirable width across the entire lumpectomy cavity. In addition, the rate of false-positive readings observed in this study compares favorably with the false-positive rate of standard histopathology. We previously found that lumpectomy margins positive on standard histopathology show no residual tumor on re-excision 65% of the time [24].

A limitation of the LUM Imaging System is its incompatibility with the blue dyes used for sentinel node mapping. Isosulfan blue and methylene blue fluoresce at wavelengths similar to those used to excite LUM015, producing high levels of signal even in areas without visible blue discoloration. We found no interference from Tc-99 sulfur colloid node mapping. Study protocols allowed the surgeon to forgo use of the LUM Imaging system and use blue dye for sentinel node mapping if radioactive signal in the axilla was not adequate, but this was not required in our study. Additional studies are under way to evaluate injection of blue dyes for sentinel node mapping after completion of the Lumicell-guided cavity assessment.

To date, there has been a single anaphylactic reaction to LUM015 among approximately 425 patients receiving intravenous LUM015 [22]. In the current study, we found no systematic changes in levels of total complement, histamine or tryptase after LUM015 injection. Additional safety testing is underway in ongoing trials.

Multicenter randomized trials are under way to further evaluate the performance of the LUM Imaging System in breast cancer surgery. Studies are under way to determine how much of the tumor left behind after standard histopathology analysis can be identified by the Lumicell system, whether the Lumicell allows removal of less normal tissue to achieve acceptable margins. Patient reported outcome measures (PROMS) are included to address patient perspectives with and without use of the LUM Imaging System. Activation of LUM015 takes place around many cancers, including tumors of the peritoneal surface [25], prostate, central nervous system and sarcomas, and is being studied in primary ovary, colorectal, esophagus and pancreas tumors.

## Data Availability

The data that support the findings of this study are not available due to them containing information that could compromise research participant privacy.
